# Supplementary material for: Geographic and Temporal Variability of Hepatitis E Virus Circulation in the Russian Federation
Source: Viruses. 2022 Dec 22;15(1):37. doi: 10.3390/v15010037 (PMC9865877; doi:10.3390/v15010037)
Supplement: Supplementary file 1 [file viruses-15-00037-s001.zip › viruses-2064477-SI.pdf]

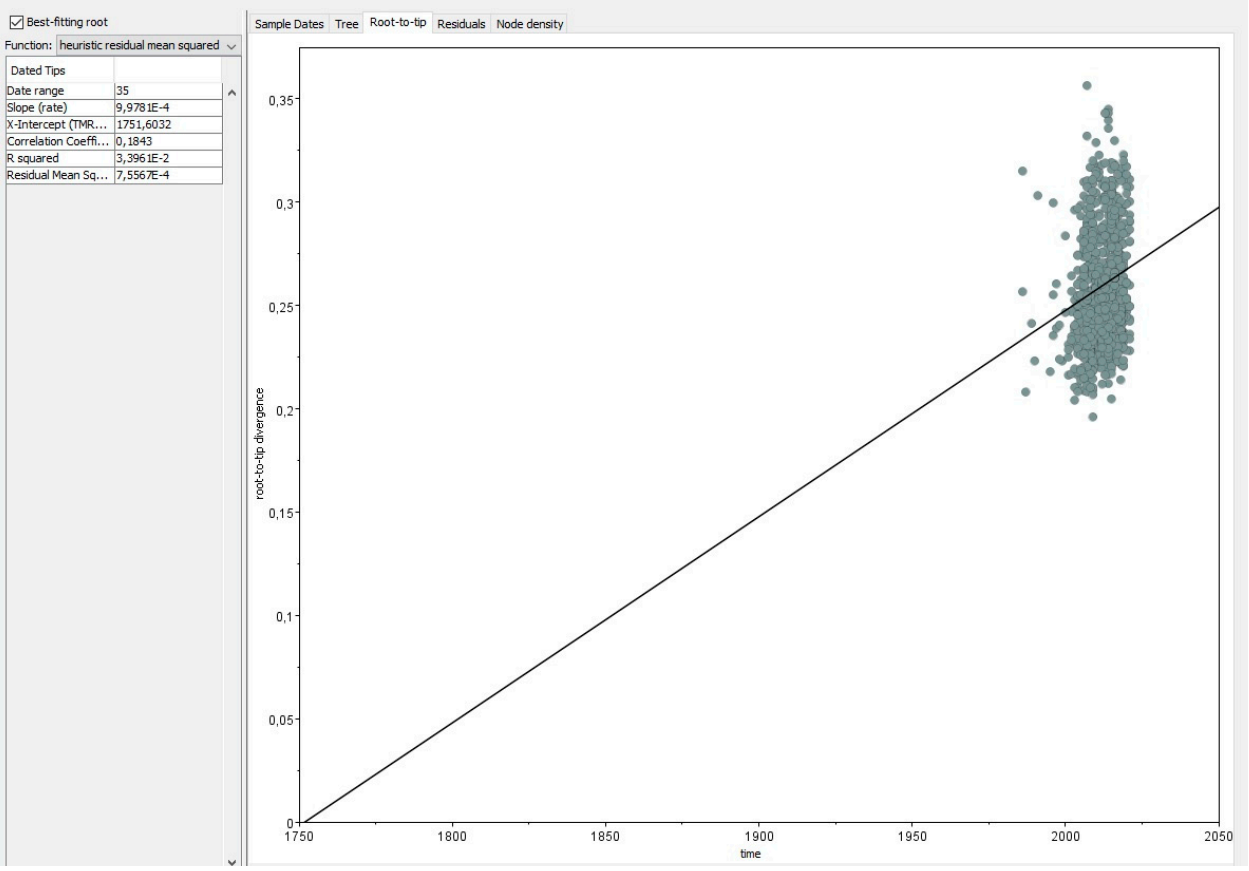


Supplementary figure S1. Temporal signal linear regression graph for HEV-3 ORF-2 300 nt fragment.

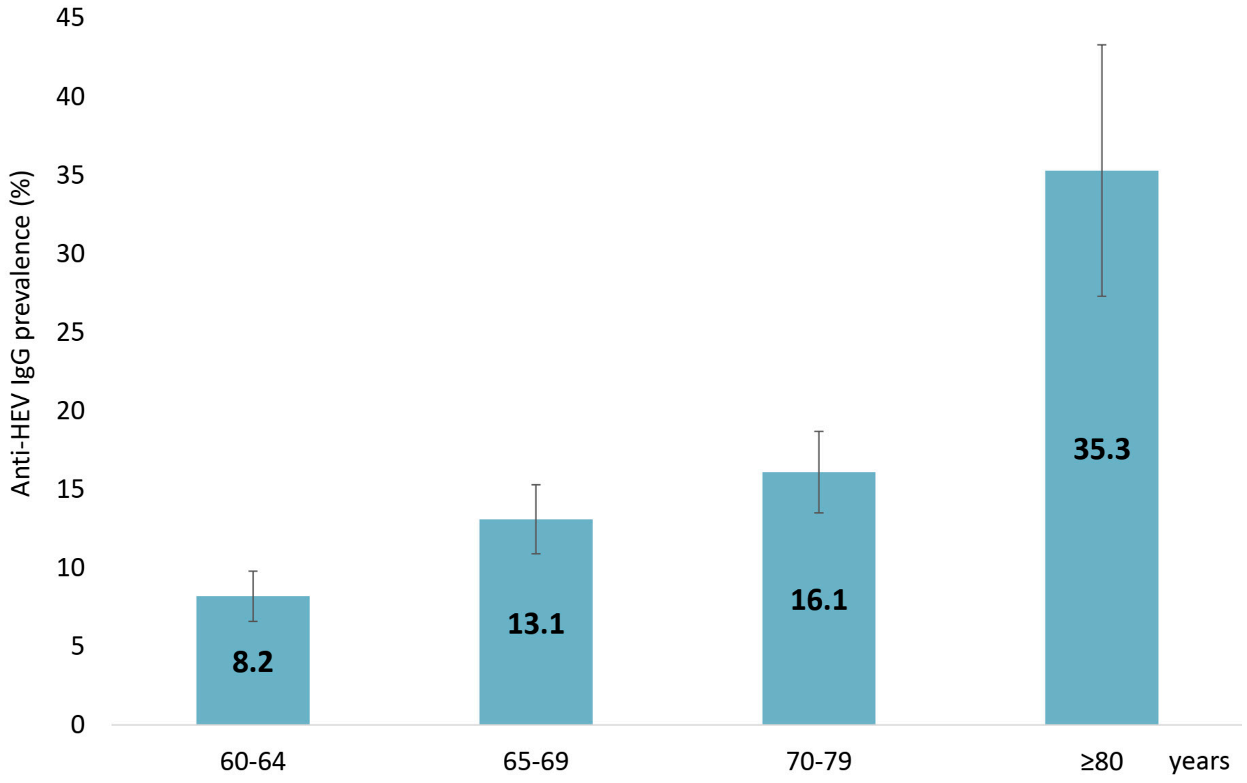


Supplementary figure S2. Age-specific anti-HEV IgG antibody prevalence rates in elderly participants from the regions studied in 2018-2020. Error bars on the bar graphs represent 95% CI.

Supplementary table S1. Anti-HEV IgG antibody positivity rates in age cohorts of healthy volunteers in regions of the Russian Federation

| Region | Year | Anti-HEV IgG<br>N positive/N tested (%) in age cohorts | | | | | | |
|---|---|---|---|---|---|---|---|---|
| | | 1-14 years | 15-19 years | 20-29 years | 30-39 years | 40-49 years | 50-59 years | ≥ 60 years |
| Kaliningrad Region | 2019 | 1/399 (0.3%) | 3/104 (2.9%) | 5/103 (4.9%) | 12/129 (9.3%) | 17/116 (14.7%) | 17/99 (17.2%) | 25/100 (25.0%) |
| St. Petersburg | 2020 | 3/467 (0.6%) | 2/129 (1.6%) | 40/1455 (2.7%) | 82/1961 (4.2%) | 39/669 (5.8%) | 21/401 (5.2%) | 23/246 (9.3%) |
| Belgorod Region | 2019 | 1/159 (0.6%) | 11/193 (5.7%) | 7/124 (5.6%) | 9/135 (6.7%) | 17/121 (14.1%) | 26/132 (19.7%) | 48/134 (35.8%) |
| Dagestan Republic | 2020 | 31/1985 (1.6%) | 8/545 (1.5%) | 6/755 (0.8%) | 6/550 (1.1%) | 7/277 (2.5%) | 10/307 (3.3%) | 135/530 (25.2%) |
| Moscow Region | 2008 | 19/455 (4.2%) | 4/152 (2.6%) | 3/104 (2.9%) | 5/100 (5.0%) | 6/105 (5.7%) | 10/114 (8.8%) | 42/149 (28.2%) |
| | 2020 | 3/714 (0.4%) | 4/212 (1.9%) | 56/2012 (2.8%) | 110/2751 (4.0) | 60/1044 (5.7%) | 40/490 (8.2%) | 24/268 (9.0%) |
| Tatarstan Republic | 2020 | 4/271 (1.5%) | 6/93 (6.5%) | 15/103 (14.6%) | 21/112 (18.8%) | 14/108 (13.0%) | 13/111 (11.7%) | 18/119 (15.1%) |
| Sverdlovsk Region | 2008 | 2/421 (0.5%) | 1/100 (1.0%) | 1/81 (1.2%) | 4/106 (3.8%) | 5/104 (4.8%) | 5/107 (4.7%) | 9/108 (8.3%) |
| | 2021 | 5/233 (2.2%) | 0/204 (0.0%) | 1/51 (2.0%) | 11/147 (7.5%) | 7/102 (6.9%) | 8/99 (8.1%) | 10/99 (10.1%) |
| Tuva Republic | 2008 | 3/388 (0.8%) | 2/100 (2.0%) | 3/100 (3.0%) | 9/100 (9.0%) | 6/100 (6.0%) | 12/100 (12.0%) | 19/123 (15.5%) |
| | 2019 | 7/359 (1.9%) | 4/167 (2.4%) | 4/147 (2.7%) | 7/169 (4.1%) | 7/112 (6.3%) | 19/108 (17.6%) | 20/99 (20.2%) |
| Novosibirsk Region | 2020 | 16/2096 (0.8%) | 13/913 (1.4%) | 40/1602 (2.5%) | 35/1615 (2.2%) | 22/829 (2.7%) | 22/519 (4.2%) | 79/757 (10.4%) |
| Sakha Republic (Yakutia) | 2008 | 2/406 (0.5%) | 0/100 (0.0%) | 2/123 (1.6%) | 1/100 (1.0%) | 3/100 (3.0%) | 2/99 (2.0%) | 11/97 (11.3%) |
| | 2018 | 7/402 (1.7%) | 2/99 (2/0%) | 8/117 (6.8%) | 6/119 (5.0%) | 4/101 (4/0%) | 12/116 (10.3%) | 13/118 (11.0%) |
| Khabarovsk Region | 2008 | 2/397 (0.5%) | 3/99 (3.0%) | 1/101 (1.0%) | 3/99 (3.0%) | 2/100 (2.0%) | 3/100 (3.0%) | 8/99 (8.1%) |
| | 2020 | 17/1770 (1.0%) | 13/527 (2.5%) | 24/713 (3.4%) | 27/624 (4.3%) | 15/294 (5.1%) | 19/333 (5.7%) | 41/509 (8.1%) |

Supplementary table S2. HEV RNA detection rates in piglets aged 2-4 months

| Region | Farm # | Number of faecal samples | Number of HEV RNA positive samples (%) |
|---|---|---|---|
| Kaliningrad | 1 | 57 | 5 (8.77%) |
| | 2 | 64 | 28 (43.75%) |
| | 3 | 76 | 0 (0.00%) |
| | 4 | 60 | 0 (0.00%) |
| Arkhangelsk | 1 | 37 | 9 (24.32%) |
| | 2 | 81 | 19 (23.46%) |
| | 3 | 137 | 24 (17.52%) |
| Belgorod | 1 | 69 | 5 (7.25%) |
| | 2 | 107 | 4 (3.74%) |
| | 3 | 101 | 50 (49.50%) |
| | 4 (2012) | 74 | 17 (22.97%) |
| | 4 (2013) | 75 | 19 (25.33%) |
| | 4 (2016) | 100 | 20 (20.00%) |
| Vladimir | 1 | 43 | 26 (60.47%) |
| Saratov | 1 | 99 | 7 (7.07%) |
| | 2 | 92 | 28 (30.43%) |
| | 3 | 91 | 45 (49.45%) |
| Sverdlovsk | 1 | 52 | 20 (38.46%) |
| | 2 | 100 | 5 (5.00%) |
| | 3 | 82 | 5 (6.10%) |
| Khabarovsk | 1 | 128 | 26 (20.31%) |
| | 2 | 107 | 0 (0.00%) |
| | 3 | 84 | 2 (2.38%) |
